# Pro-dermcidin and derivatives as potential therapeutics for lethal experimental sepsis

**DOI:** 10.3389/fimmu.2025.1621633

**Published:** 2025-06-04

**Authors:** Weiqiang Chen, Xiaoling Qiang, Cassie Shu Zhu, Jianhua Li, Li Lou, Ping Wang, Kevin J. Tracey, Haichao Wang

**Affiliations:** ^1^ The Feinstein Institutes for Medical Research, Northwell Health, Manhasset, NY, United States; ^2^ Departments of Emergency Medicine and/or Molecular Medicine, Donald and Barbara Zucker School of Medicine at Hofstra/Northwell, Hempstead, NY, United States

**Keywords:** innate immune cells, pro-dermcidin, PEGylation, anti-bacterial, anti-inflammation, LC3 activation

## Abstract

A 110-amino acid precursor of dermcidin (pre-dermcidin, pre-DCD) with a 19-residue N-terminal leader signal sequence can be secreted by human eccrine sweat glands as a leader-less pro-domain-containing peptide (pro-DCD), which is enzymatically cleaved to generate C-terminal anti-microbial peptides (dermcidin-1, DCD-1) capable of killing various bacteria. Previously, it was unknown whether pro-DCD could be pharmacologically developed as potential therapeutics for lethal sepsis. Here, we demonstrated that pharmacological suppression of pro-DCD with polyclonal antibodies worsened sepsis-induced inflammation and liver injury, whereas supplementation of pro-DCD or its PEGylation derivatives significantly protected against sepsis, even when given 2–24 h after disease onset. These protective effects were associated with a significant reduction in circulating levels of surrogate biomarkers [e.g., Granulocyte Colony Stimulating Factor (G-CSF), Interleukin-6 (IL-6), keratinocytes-derived chemokine (KC), Monocyte Chemoattractant Protein 1 (MCP-1), Macrophage Inflammatory Protein-2 (MIP-2), and Soluble Tumor Necrosis Factor Receptor I (sTNFRI)], tissue injury, and blood bacterial counts. Although pro-DCD or its PEGylation derivatives failed to directly kill bacteria across a wide range of concentrations, they were able to activate microtubule-associated protein 1A/1B-light chain 3 (LC3), a marker of autophagy and phagosome maturation in LC3-associated bacterial phagocytosis. Our findings suggest that pro-DCD-derived agents hold promise as potential therapies for clinical sepsis.

## Introduction

Throughout evolution, primates and humans have developed eccrine sweat glands that produce sweat as an efficient mechanism for evaporative heat loss and thermoregulation. In humans, these eccrine sweat glands also express various antibiotic peptides, including pre-dermcidin (pre-DCD) (1), a 110-amino acid precursor protein with a 19-residue N-terminal leader signal sequence. Once this leader signal sequence is removed, the leader-less pro-dermcidin (pro-DCD) is secreted by the eccrine sweat glands and enzymatically cleaved to remove the pro-domain to produce the mature form dermcidin (DCD), which can kill several bacteria such as *Escherichia coli*, *Enterococcus faecalis*, and *Staphylococcus aureus* (2).

In addition to eccrine sweat glands (1, 2), there is evidence (2) that pro-DCD may also be up-regulated and/or secreted by other cell types, such as monocytes (3), bone marrow-derived mesenchymal stromal cells (BM-MSCs) (4), and skeletal muscles (5) in response to various challenges, including HIV infection (3), *E. coli* exposure (4), or facioscapulohumeral muscular dystrophy (FSHD) (5). Consistently, pro-DCD has been detected in the blood of patients with diverse conditions such as ischemic stroke (4), FSHD (5), melanoma (6), and obstructive sleep apnea (7). Even in rodents, there is some evidence for the possible expression of pro-DCD in the sweat gland of the footpad (8), peripheral blood mononuclear cells (PBMCs) (8), and skeletal muscles in both healthy rats (8) and murine models of myocardial ischemia/reperfusion (9). Previously, it was entirely unknown whether pro-DCD could be developed into potential therapeutics for lethal sepsis. Here, we presented compelling evidence to support a therapeutic potential of pro-DCD and PEGylation derivatives in pre-clinical model of sepsis by attenuating dysregulated inflammation and possibly facilitating LC3-associated phagocytosis and/or autophagic degradation of engulfed pathogens.

## Materials and methods

### Materials

Murine macrophage RAW 264.7 cell line was purchased from the American Type Culture Collection (ATCC). Macrophage cultures were routinely cultured in DMEM media containing 1% streptomycin/penicillin and 10% fecal bovine serum. When cell densities reached 80-90% confluence, adherent macrophages were cultured in serum-free OPTI-MEM I medium and stimulated with recombinant human pro-DCD-C34S. The intracellular levels of LC3-I and lipidated LC3-II were respectively measured by Western blotting using specific antibodies.

### Western blotting

The concentrations of cellular LC3-I and lipidated LC3-II in pro-DCD-stimulated murine macrophage-like RAW 264.7 cells were measured by Western blotting using rabbit anti-mouse LC3A/B monoclonal antibodies (Cat. # 12741, Cell Signaling) as previously described (10). Briefly, equal volume of cell-conditioned culture medium or murine/human serum were resolved on sodium dodecyl sulfate (SDS)-polyacrylamide gels and transferred to polyvinylidene difluoride (PVDF) membranes. After blocking with 5% nonfat milk, the membranes were incubated with the appropriate antibodies (anti-pro-DCD, 1:1000; anti-LC3A/B, 1:1000) overnight. Subsequently, the membranes were incubated with secondary antibodies (mouse-anti-rabbit IgG-HRP, Cat.# sc-2357, Santa Cruz; or donkey-anti-rabbit IgG-HRP, Cat. # NA934, GH Healthcare), and the immune-reactive bands were visualized by chemiluminescence. The relative levels of specific proteins were determined using the UN-SCAN-IT Gel Analysis Software Version 7.1 (Silk Scientific Inc., Orem, UT, USA) and expressed in arbitrary units (AU).

### Preparation of recombinant human pro-DCD and pro-DCD-C34S proteins

The cDNA encoding human pro-dermcidin (residue 20-110) or a mutant with a Cys (C)→Ser (S) substitution at residue 34 (pro-DCD-C34S) was cloned into a pReceiver expression vector downstream of a T7 promoter with an N-histidine tag, and expressed in *E. coli* BL21 (DE3) pLysS cells as previously described (11, 12). The inclusion body-associated recombinant pro-DCD or pro-DCD-C34S protein was isolated by differential centrifugation and urea solubilization, followed by refolding in Tris buffer (pH 8.0) containing N-lauroylsarcosine. The recombinant pro-DCD and pro-DCD-C34S were further purified using histidine-affinity chromatography, followed by extensive Triton X-114 extractions to remove contaminating endotoxins. The recombinant pro-DCD or pro-DCD-C34S protein were tested for LPS content by the chromogenic *Limulus* amebocyte lysate assay (Endochrome; Charles River), and the endotoxin content was less than 0.01 U per microgram of recombinant protein.

### Generation of anti-pro-DCD polyclonal antibodies

Polyclonal antibodies were generated in Female New Zealand White Rabbits by the Covance Inc. (Princeton, NJ, USA) using recombinant human pro-DCD combined with Freund’s complete adjuvant following standard procedures. Blood samples were collected in 3-week cycles of immunization and bleeding, and the antibody titers were determined by direct pro-DCD ELISA. Total IgGs and pro-DCD antigen-binding IgGs were purified from the anti-pro-DCD rabbit serum using Protein A and pro-DCD-affinity column chromatography, respectively. Briefly, rabbit serum was pre-buffered with PBS and slowly loaded onto the Protein A/G Sepharose (Cat. # ab193262) column to allow sufficient binding of IgGs. After washing with 1×PBS to remove unbound serum components, the IgGs were eluted with acidic buffer (0.1 M glycine-HCl, pH 2.8), and then immediately dialyzed into 1×PBS buffer at 4°C overnight. For pro-DCD antigen-affinity purification, recombinant human pro-DCD was conjugated to cyanogen bromide (CNBr)-activated Sepharose4 agarose beads (Cat. # 17098101, GE Healthcare), which were then loaded into columns. After repeated washings with acid buffer (0.1 M Acetic/Sodium Acetate, 0.5 M NaCl, pH 4.0) and alkali buffer (0.1 M Tris-HCl, 0.5 NaCl, pH 8.0), anti-pro-DCD total IgGs were slowly loaded onto the column, and the flow-through fractions were collected. After repetitive washing with 1×PBS buffer, the pro-DCD-binding antibodies were eluted with acidic elution buffer, and immediately neutralized in 1×PBS solution.

### Peptide dot blotting

A library of 13 synthetic peptides corresponding to various regions of human pro-DCD sequence was synthesized at the Genscript, and spotted (0. 1 μg in 2.5 μl) onto a nitrocellulose membrane (Thermo Scientific, Cat No. 88013). Subsequently, the membrane was probed with anti-pro-DCD rabbit serum or pro-DCD antigen affinity-purified IgGs, following a standard protocol as previously described (13, 14).

### Cytokine Antibody Arrays

Murine Cytokine Antibody Arrays (Cat. No. M0308003, RayBiotech Inc., Norcross, GA, USA), which simultaneously detect 62 cytokines on one membrane, were used to measure relative cytokine concentrations in murine serum as described previously (13–15).

### PEGylation of pro-DCD-C34S

Recombinant pro-DCD-C34S was pegylated using methyl-PEG_24_-NHS ester (MW of 1214.39 Daltons, Cat.# 22687, Thermo Scientific, Rockford, IL, USA) according to manufacturer’s instructions. Briefly, methyl-PEG_24_-NHS ester was dissolved in DMSO (5.0 mg/ml) and mixed with a solution of pro-DCD-C34S at a 20-fold molar excess to the protein, ensuring the final DMSO concentration was below 10%. After incubating the mixture on ice for two hours, the solution was dialyzed overnight at 4° C in 1×PBS buffer to remove any unreacted PEG NHS ester.

### Animal model of experimental sepsis

Every effort was made to minimize the number of animals used in this study in accordance with the ARRIVE guidelines developed by the British National Centre for the Replacement, Refinement and Reduction of Animals in Research (NC3Rs). Additionally, all experiments were conducted following the International Expert Consensus Initiative for Improvement of Animal Modeling in Sepsis - Minimum Quality Threshold in Pre-Clinical Sepsis Studies (MQTiPSS) (16). This includes practices such as randomizing animals within each experimental group, implementing delayed therapeutic interventions (with agents like pegylated pro-DCD-C34S) (17), establishing specific criteria for euthanizing moribund septic animals (e.g., labored breathing, minimized response to human touch, and immobility), and administering fluid resuscitation and antibiotics (18). This study received administrative approval from the IACUC of the Feinstein Institutes for Medical Research (FIMR, Protocol # 2017–038 Term II; Date of Approval, February 22, 2021).

Adult male and female Balb/C mice (7–8 weeks old, 20–25 g body weight) were purchased from Charles River Laboratories (Wilmington, MA), and acclimated for at least 5–7 days before usage. To induce experimental sepsis, Balb/C mice underwent a surgical procedure known as “cecal ligation and puncture” (CLP) as previously described (13, 14, 19). Prior to CLP surgery, all animals received a buprenorphine injection (0.05 mg/kg, subcutaneously) to manage immediate surgical pain, because repetitive use of buprenorphine in the CLP model could paradoxically elevate sepsis surrogate markers and increase animal lethality (20, 21). Approximately 30 min post CLP surgery, all experimental animals were subcutaneously injected with a dose of imipenem/cilastatin (0.5 mg/mouse) (Primaxin, Merck & Co., Inc.) to avoid their potential adverse effects on the therapeutic efficacy of pro-DCD-C34S or derivatives. Animals were randomly assigned to control vehicle and experimental groups, and pro-DCD, pro-DCD-C34S, or pegylated pro-DCD-C34S was intraperitoneally injected to septic mice at various time points post-CLP. Animal survival was monitored for two weeks to ensure no late death occurred. To elucidate the potential protective mechanisms of peg-pro-DCD-C34S, a separate group of Balb/C mice underwent CLP and received peg-pro-DCD-C34S (0.2 mg/kg) at 2 h and 20 h post-CLP. At 24 h post CLP, animals were euthanized to collect blood and measure: i) serum levels of various cytokines and chemokines using murine Cytokine Antibody Arrays; ii) markers of liver injury using specific colorimetric enzymatic assays; and iii) blood bacterial colony forming units as previously described (13).

Male and female mice were not mixed in the same experiments to avoid potential influences of female hormones on the host immune response to infections. Instead, all experiments were initially conducted using male mice and subsequently replicated in age-matched female mice in separate studies to assess potential gender-specific differences. When no gender-specific differences were observed, the results from both male and female mice were pooled to reduce the number of animals used while ensuring robust and reliable conclusions.

### Measurement of liver injury markers

Blood samples were collected at 24 h post-CLP following intraperitoneal administrations of pegylated pro-DCD-C34S (0.2 mg/kg) at 2 h and 20 h post-CLP. The samples were centrifuged at 3000 x g for 10 min to collect serum, and serum levels of liver injury markers such as aspartate aminotransferase (AST, Cat. No. 7561) and alanine aminotransferase (ALT, Cat. No. 7526) were measured using specific colorimetric enzymatic assays (Pointe Scientific, Canton, MI) according to manufacturer’s instructions as previously described (13, 22).

### Liver histological analysis

The liver samples were collected at 6 h post CLP, fixed in 10% buffered formalin, and embedded in paraffin. The paraffin-embedded tissues were sectioned into 5-μm slices, stained with hematoxylin-eosin, and examined under light microscopy. Liver parenchymal injury was assessed in a blinded fashion by summing three different Suzuki scores (ranging from 0-4) for sinusoidal congestion, hepatocyte cytoplasmic vacuolization, and parenchymal necrosis as previously described (12, 14). The parameter scores were calculated using a weighted equation with a maximum score of 100 per field and then averaged to determine the final liver injury score for each experimental group.

### Evaluation of bactericidal activity

*Escherichia coli* BL21 (DE) strain was inoculated into Luria Bertani Broth (LB broth), incubated at 37°C until the culture reached the mid-log phase. Pro-DCD or peg-pro-DCD-C34S was dissolved in 1 × PBS buffer at various dilutions and mixed with the *E. coli* suspension in 1 × PBS. The mixture was incubated at 37°C for 4 h in sterile test tubes as previously described (1, 23). After incubation, the bacterial suspension was serially diluted in sterile 1 × PBS solution and spread onto LB agar plates. Following incubation 37°C for 16–24 h, the number of colony-forming units (CFUs) on each plate was counted to determine whether pro-DCD or its derivatives reduced the CFU count of the *E. coli* suspension.

### Visualization of LC3-positive punctate structures

Murine macrophage-like RAW 264.7 cells stably transfected with GFP-LC3 were stimulated with pro-DCD (1.0 µg/ml) for 16 h, and the formation of GFP-LC3 punctate structures was examined under a fluorescence microscope as previously described (10). The ratio between the 18-kD cytosolic LC3-I and 16-kD lipidated LC3-II was determined by Western blotting analysis following previously described methods (10).

### Statistical analysis

All data were first assessed for normality using the Shapiro-Wilk test before applying the appropriate statistical analyses. The Student’s t test was employed to compare two independent experimental groups. For comparison among multiple groups with non-normal (skewed) distribution, the Kruskal-Wallis ANOVA test followed by Dunn’s *post hoc* test was used to evaluate statistical differences. The Kaplan-Meier method, along with the nonparametric log-rank test, was used to compare mortality rates between different groups. Statistical significance was defined as a *P* value less than 0.05.

## Results

### Generation of human pro-DCD-specific polyclonal antibodies

**Figure 1:**
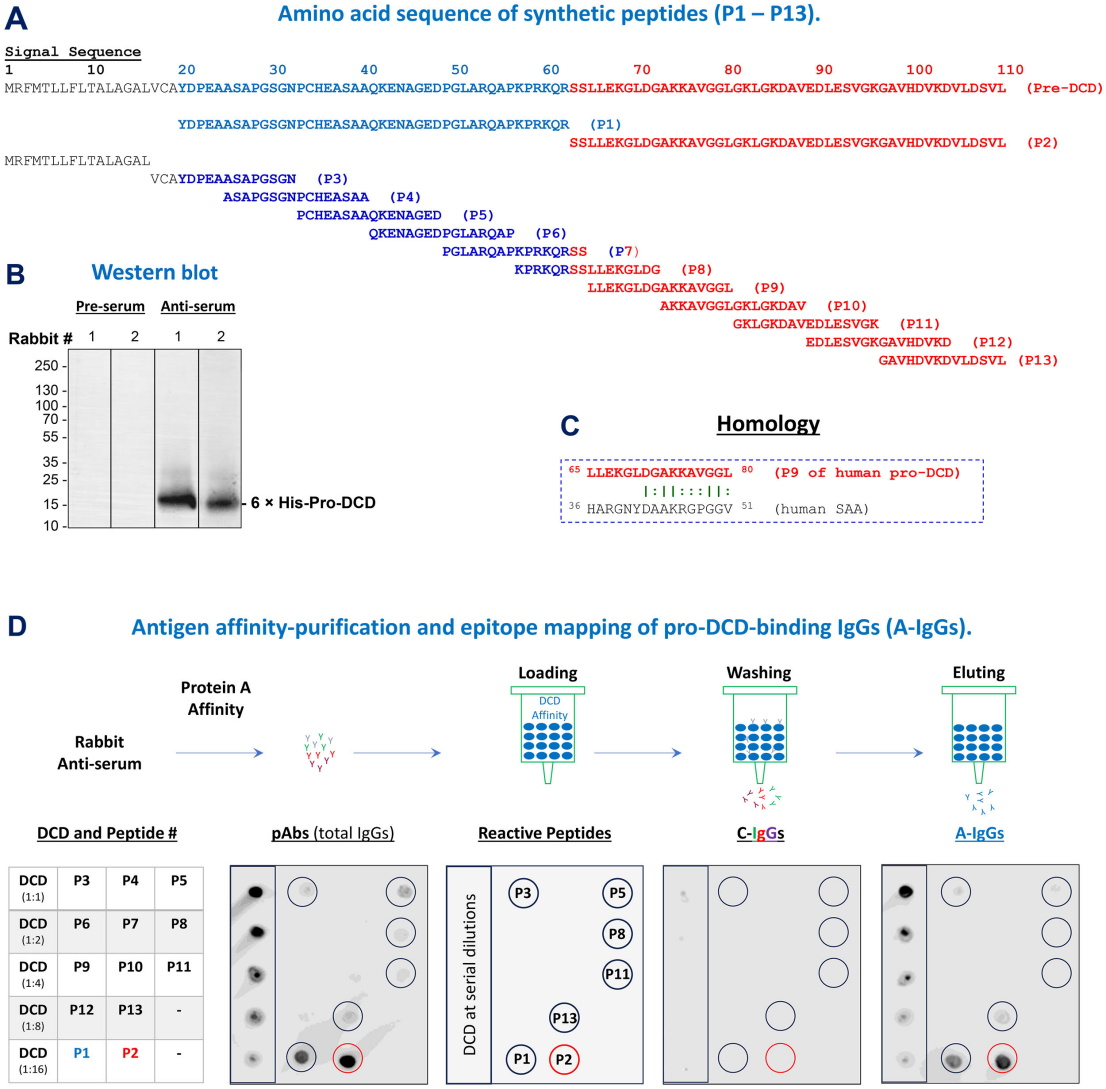
Generation and epitope mapping of human pro-DCD-specific polyclonal antibodies. **(A)** Sequence of 13 synthetic peptides corresponding to various regions of human pro-DCD. **(B)** Western blotting analysis comparing pre-immune and anti-pro-DCD serum from two representative rabbits. **(C)** Depiction of a peptide sequence (P9) that exhibits homology to an inducible human acute-phase protein, serum amyloid A (SAA), with a similar molecular weight **(D)** Schematic representation of antigen affinity-purification and epitope mapping of anti-pro-DCD IgGs (“A-IgGs”). Total IgGs were purified from anti-human pro-DCD rabbit serum using Protein A affinity chromatography, followed by the isolation of pro-DCD-binding IgGs (“A-IgGs”) through pro-DCD antigen-affinity chromatography. Non-pro-DCD-binding control immunoglobulins (“C-IgG”) were collected from the washout fractions, while the pro-DCD antigen-bound antibodies (“A-IgGs”) were eluted from the column using an acidic buffer, and then neutralized to a physiological pH to prevent acid-catalyzed antibody denaturation. Epitope mapping was conducted by dot blotting analysis of recombinant pro-DCD and the 13 synthetic peptides corresponding to different region of pro-DCD.

To understand the extracellular role of pro-DCD in sepsis, we synthetized thirteen peptides corresponding to different regions of human pro-DCD (**Figure 1A**) and used them to profile the epitopes of rabbit polyclonal antibodies that we raised against human pro-DCD (**Figure 1B**). As predicted, immunoblotting of recombinant pro-DCD with pre-immune rabbit serum did not reveal any immune-reactive band (**Figure 1B**), indicating that these rabbits did not produce pro-DCD-reactive autoantibodies. However, Western blotting with anti-pro-DCD serum revealed a strong band corresponding to the molecular weight of recombinant pro-DCD with an N-terminal 6×His tag (11, 12) (**Figure 1B**). Consistently, our home-made pro-DCD protein was recognized by some commercial polyclonal antibodies (ab175519) raised against a unique peptide sequence (residue 96-110) of human pro-DCD (**Supplementary Figure S1**).

To obtain pro-DCD antigen-affinity purified total IgGs (“A-IgGs”), we first subjected anti-pro-DCD rabbit serum to Protein-A-affinity chromatography to obtain total IgGs (“pAbs”, **Figure 1D**, Top Panel), followed by pro-DCD-antigen-affinity chromatography to isolate the antigen-binding IgGs (“A-IgGs”) (**Figure 1D**, Top Panel). Dot blotting analyses demonstrated that these anti-pro-DCD pAbs were reactive with recombinant pro-DCD at various dilutions, as well as several synthetic peptides (e.g., P1, P2, P3, P5, P8, P11 and P13) corresponding to different regions of pro-DCD (**Figure 1D**), confirming their specificity for human pro-DCD. Additionally, the epitope profiles of pro-DCD antigen-affinity purified IgGs (“A-IgGs”) were consistent with those of the crude anti-pro-DCD serum (**Figure 1D**), validating the specificity of our home-made anti-pro-DCD pAbs to multiple pro-DCD peptides, including epitopes corresponding to residue 97–110 of pro-DCD (i.e., “P13”, **Figure 1D**). Despite some homology between two short peptides of pro-DCD (residue 65-80 or “P9”) and the acute phase protein serum amyloid A (SAA, residue 36-51, **Figure 1C**), our anti-pro-DCD pAbs did not react with the P9 peptide, suggesting that our home-made anti-pro-DCD pAbs were unlikely to cross-react with human SAAs.

### Pro-DCD-reactive polyclonal antibodies worsened the outcome of experimental sepsis

**Figure 2:**
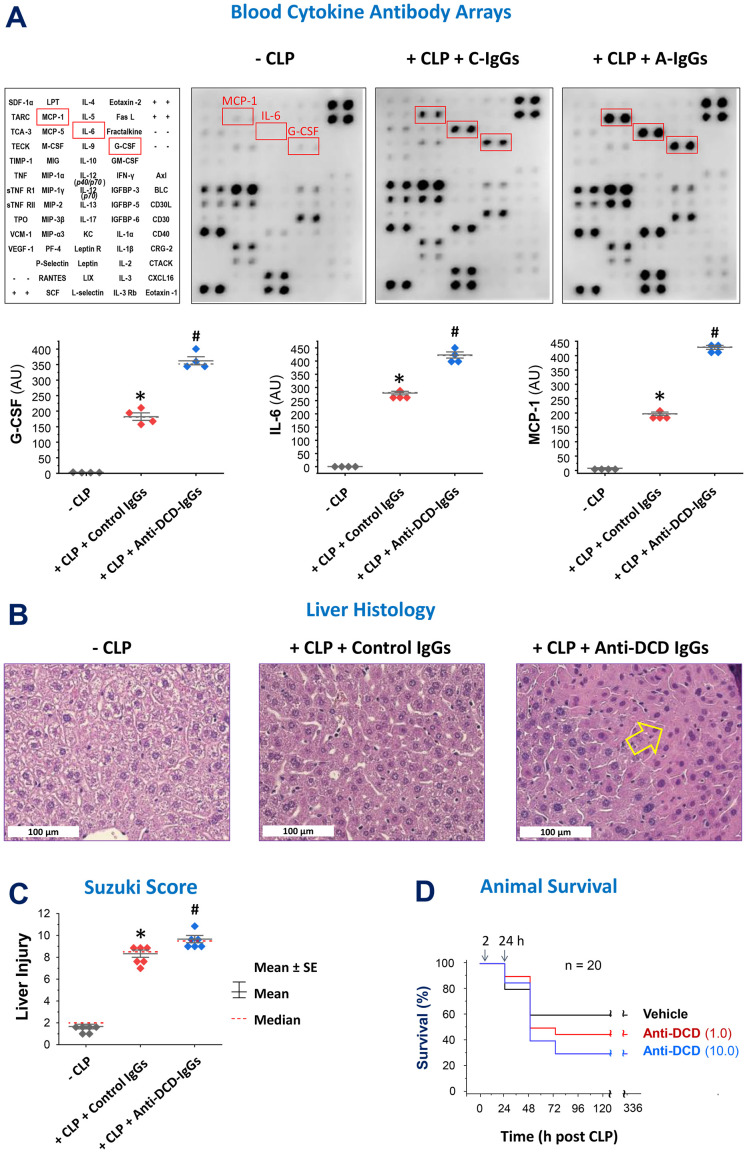
Pro-DCD-reactive IgGs worsened sepsis-induced inflammation and tissue injury in mice. **(A–C)** Pro-DCD-specific IgGs aggravated sepsis-induced inflammation and tissue injury. Balb/C mice were subjected to CLP-induced experimental sepsis, followed by intraperitoneal administration of either pro-DCD-non-reactive control antibodies (“C-IgGs”) or antigen-affinity purified anti-pro-DCD IgGs (“A-IgGs”, 10 mg/kg) at 2 h post-CLP. At 6 h post-CLP, the animals were sacrificed to harvest blood and liver tissues to measure blood levels of various cytokines and chemokines using Cytokine Antibody Arrays **(A)** and assess liver injury through histological analysis **(B)** and assessment of Suzuki Injury Scores **(C)**. *, *P* < 0.05 vs. negative control ("-CLP"); #, *P* < 0.05 vs. positive control ("Control IgGs"). non-parametric Kruskal-Wallis ANOVA test. **(D)** Antigen affinity-purified anti-DCD antibodies (“A-IgGs”) appeared to increase sepsis-induced mortality in a dose-dependent manner.

To explore the role of pro-DCD in experimental sepsis, we assessed the impact of antigen affinity-purified anti-pro-DCD IgGs on sepsis-induced systemic inflammation and tissue injury. Notably, pro-DCD-specific IgGs (“A-IgGs”) significantly elevated sepsis-induced systemic accumulation of G-CSF, IL-6 and MCP-1 (**Figure 2A**), three surrogate markers of experimental (24, 25) and clinical sepsis (26). Consistently, these anti-pro-DCD antibodies exacerbated sepsis-induced liver injury (**Figures 2B, C**) and appeared to dose-dependently increase sepsis-induced animal lethality (**Figure 2D**). Collectively, these findings have suggested a potentially beneficial role of pro-DCD in an animal model of experimental sepsis.

### Pro-DCD and PEGylation derivatives conferred a significant protection against lethal sepsis

**Figure 3:**
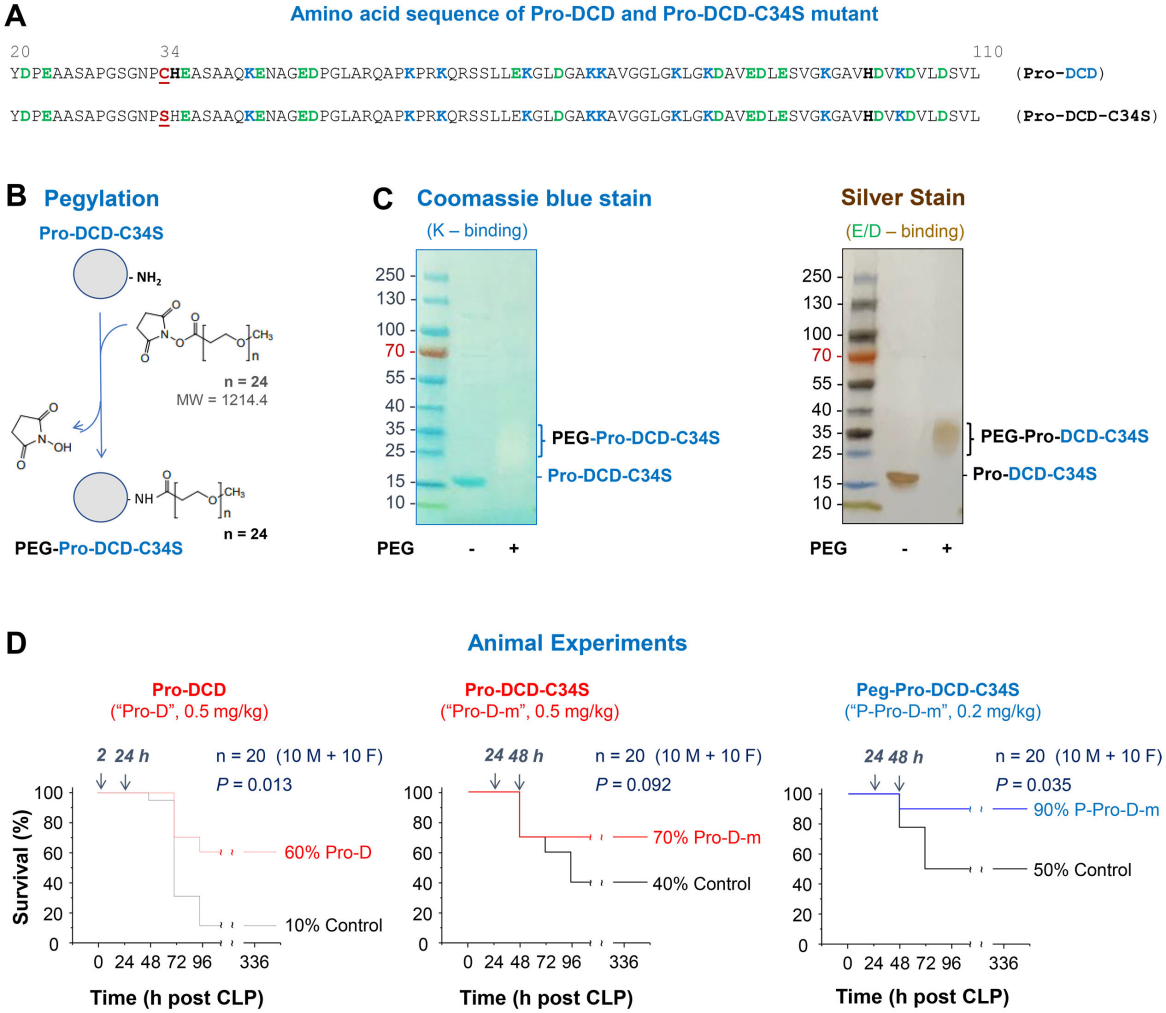
Generation of pro-DCD-C34S mutant and PEGylation derivatives for treating experimental sepsis. **(A)** Amino sequence of recombinant pro-DCD mutant containing a Cys (C)→Ser (S) substitution at residue 34 (pro-DCD-C34S). **(B)** PEGylation of pro-DCD-C34S using methyl-PEG_24_-NHS ester potentially targeting ten Lys (K) residues. **(C)** Detection of pegylated pro-DCD-C34S (Peg-pro-DCD-C34S) through SDS-PAGE gel electrophoresis and silver staining. The PEG-DCD-C34S was not visible with Coomassie blue staining due to the PEGylation of amine groups of Lys (K) residues, which prevented its electrostatic interaction with the Coomassie Brilliant Blue G-250 dye. However, pegylated pro-DCD-C34S was detectable by silver staining as silver nitrate could still interact with other residues, such as the carboxylic acid groups of Asp (D) and Glu (E) or imidazole of His (H) of pro-DCD-C34S. **(D)** Pro-DCD and PEG-pro-DCD-C34S rescued mice from lethal sepsis. Balb/C mice (male or female, 8–10 weeks, 20–25 g) mice were subjected to CLP -induced sepsis, and highly purified recombinant pro-DCD, pro-DCD-C34S, or pegylated pro-DCD-C34S were administered intraperitoneally at specified doses and time points after CLP. Animals were monitored for two weeks to ensure long-lasting effects. *, *P* < 0.05 versus saline control group.

To assess the therapeutic potential of pro-DCD, we generated recombinant pro-DCD corresponding to residue 20-110 (excluding the signal leader peptide, **Figure 3A**) with an N-terminal 6×His tag as previously described (11, 12). Because recombinant pro-DCD could form dimers via disulfide bond at Cys (C) 34, we also created a pro-DCD variant with a Cys (C)→Ser (S) substitution at residue 34 (pro-DCD-C34S, **Figure 3A**), which migrated as a 16-kDa monomer on SDS-PAGE even in the absence of a reducing agent, DTT (**Figure 3C**). To extend the half-life of pro-DCD-C34S, we chemically conjugated highly hydrophilic polyethylene glycol (PEG) chains to the Lys (K) residue of this peptide (**Figure 3B**). This modification is expected to shield pro-DCD-C34S from enzymatic degradation and prevent it from renal clearance. As anticipated, pegylated pro-DCD-C34S was not detected by Coomassie blue staining (**Figure 3C**, Left Panel), because PEGylation of amine groups of Lys (K) residues obstructed its electrostatic interaction with the Coomassie Brilliant Blue G-250 dye. However, the pegylated pro-DCD-C34S was still detected by silver staining as larger molecules ranging 25 to 37 kDa (**Figure 3C**, Right Panel), since silver nitrate could still interact with other residues such as the carboxylic acid groups of Asp (D) and Glu (E) or imidazole of His (H) of pro-DCD-C34S.

After extensive extraction with Triton X-114 to remove endotoxin contaminants, we evaluated the therapeutic efficacy of highly purified pro-DCD, pro-DCD-C34S, and pegylated pro-DCD-C34S in a murine model of experimental sepsis. Administration of recombinant pro-DCD at 2 h and 24 h post-CLP significantly improved animal survival rate, increasing it from 10% in the saline control to 60% in the pro-DCD-treatment group (**Figure 3D**, Left Panel). When the 1^st^ dose was delayed to 24 h post-CLP, the pro-DCD-C34S analog showed a similar tend towards increasing animal survival at the same dose (0.5 mg/kg BW, **Figure 3D**, Middle Panel). Notably, pegylated pro-DCD-C34S provided a significant protection against lethal sepsis even when given in a delayed fashion (24 h post-CLP) at a dose (0.2 mg/kg) that was approximately 5-fold lower in molar concentration compared to pro-DCD-C34S (**Figure 3D**, Right Panel), considering the almost two-fold difference in molecular weight between pro-DCD-C34S (~15 kDa) and PEG-pro-DCD-C34S (25–37 kDa, **Figure 3C**). Collectively, these results indicate that both pro-DCD and pro-DCD-C34S derivatives offer significant protection against lethal sepsis.

### Peg-pro-DCD-C34S reduced blood levels of surrogate inflammatory and liver markers and bacterial counts

**Figure 4:**
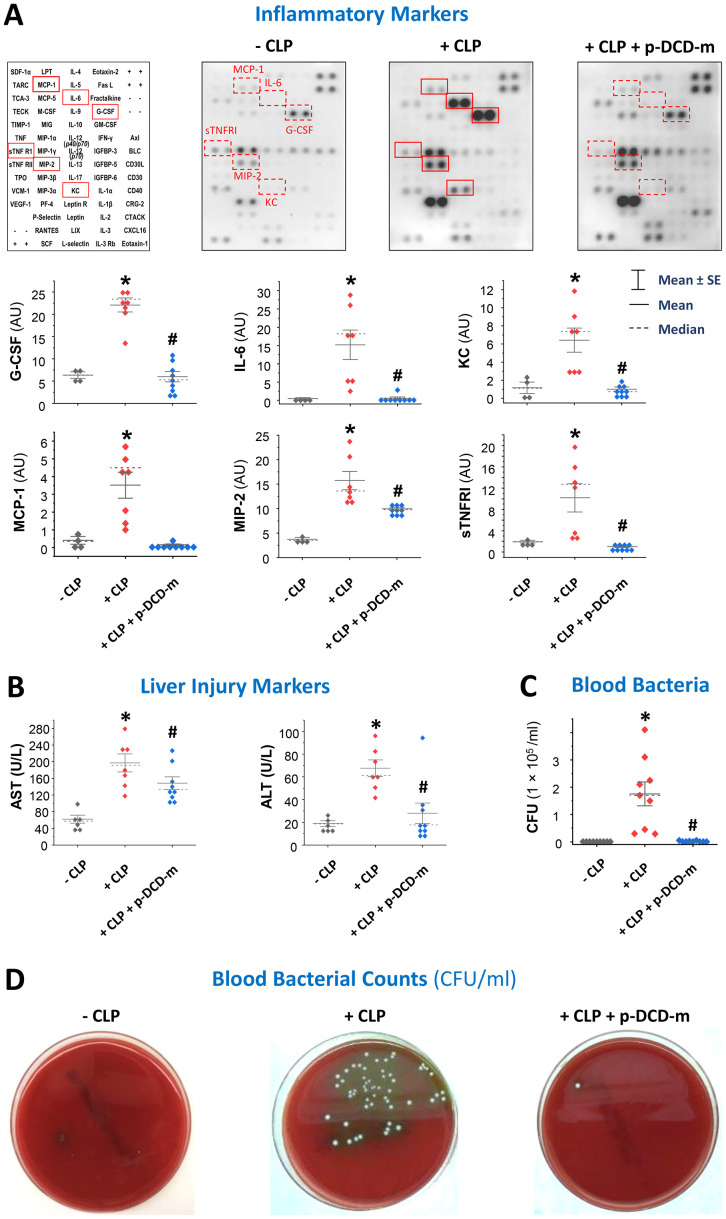
Pegylated pro-DCD-C34S significantly reduced sepsis-induced systemic inflammation, liver injury, and blood bacterial loads. **(A, B)** Pegylated pro-DCD-C34S significantly attenuated sepsis-induced systemic inflammation and liver injury. Balb/C mice were subjected to CLP-induced sepsis, and pegylated pro-DCD-C34S (0.2 mg/kg) was given twice at 2 and 20 h post-CLP. At 24 h post-CLP, animals were sacrificed to harvest blood to measure various cytokines and chemokines **(A)** and liver injury markers such as AST and ALT **(B)**. *P < 0.05 *vs*. negative control (“- CLP”); #*P* < 0.05 *vs*. positive control (“+ CLP”) group, non-parametric Kruskal-Wallis ANOVA test. **(C, D)** Pegylated pro-DCD-C34S markedly reduced blood bacterial load in septic animals. Balb/C mice were subjected to CLP-induced sepsis, and pegylated pro-DCD-C34S (0.2 mg/kg) was given twice at 2 and 20 h post-CLP. At 24 h post-CLP, animals were sacrificed to harvest blood to count bacterial colony forming unit (CFU) after serial dilution and plating on LB agar plates. *P* < 0.05 *vs*. negative control (“- CLP”); #*P* < 0.05 *vs*. positive control (“+ CLP”), non-parametric Kruskal-Wallis ANOVA test.

To investigate the mechanisms underlying pro-DCD-mediated protection, we assessed the effects of pegylated pro-DCD-C34S on sepsis-induced systemic inflammation, tissue injury, and bacterial dissemination. Intraperitoneal administration of peg-pro-DCD-C34S significantly reduced sepsis-induced systemic accumulation of G-CSF, IL-6, KC, MCP-1, MIP-2, and sTNFRI (**Figure 4A**), indicating that pro-DCD derivatives confer protection against sepsis by mitigating systemic inflammation of key sepsis surrogate biomarkers of experimental (24, 25) and clinical sepsis (26). Additionally, peg-pro-DCD-C34S significantly attenuated sepsis-induced elevation of liver enzymes such as AST and ALT (**Figure 4B**), suggesting that pro-DCD derivatives provide protection against lethal sepsis partly by alleviating both inflammation and tissue injury.

To further elucidate the protective mechanism of pro-DCD, we also examined the impact of peg-pro-DCD-C34S on blood bacterial counts. As predicted, CLP led to a substantial increase in blood bacterial levels (**Figures 4C, D**), reflecting cecal bacterial spillage into the blood stream. However, treatment with peg-pro-DCD-C34S almost completely diminished blood bacterial counts (**Figures 4C, D**), suggesting that pro-DCD conferred protection against lethal sepsis partly by facilitating bacterial clearance.

### 3 Pro-DCD failed to directly kill bacteria but induced LCactivation in macrophage cultures

**Figure 5:**
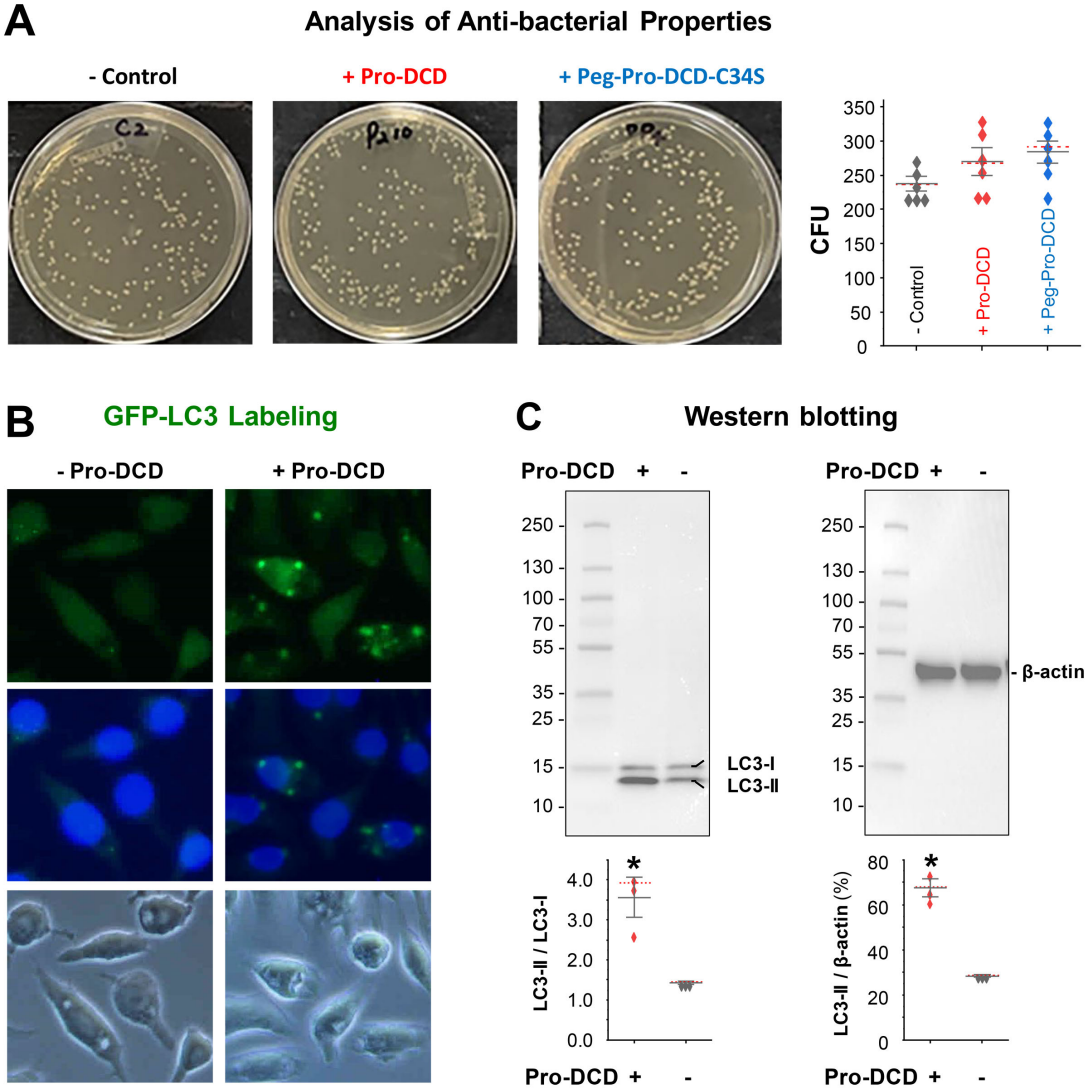
Pro-DCD failed to directly kill bacteria but significantly activated LC3 in macrophage cultures. **(A)** Pro-DCD failed to directly kill *E coli*. Pro-DCD-C34S or its pegylated derivatives were incubated with *E coli* strain at various concentrations (up to 200 µg/ml) for 4 hours, and then plated onto LB agar plates. After overnight incubation at 37°C, the number of colony forming units (CFU) was counted. **(B)** Pro-DCD-C34S induced the formation of LC3 puncta in macrophage cultures. GFP-LC3-transfected murine macrophage-like RAW 264.7 cells were stimulated with pro-DCD-C34S (0.2 µg/ml) for 16 h, and the formation of LC3 puncta was observed under fluorescent microscopy. **(C)** Pro-DCD-C34S induced LC3-II production in macrophage cultures. Macrophage-like RAW264.7 cells were stimulated with pro-DCD-C34S for 16 h, and cellular levels of LC3-I and LC3-II were determined by Western blotting analysis with reference to β-actin. *P < 0.05 versus negative control (“- pro-DCD”), non-parametric Kruskal-Wallis ANOVA test.

To understand how pro-DCD facilitates bacterial elimination in septic animals, we incubated pro-DCD or peg-pro-DCD-C34S with *E. coli* for 3–4 hours and plated the mixture onto LB broth agar at serial dilutions to count the colony forming unit (CFU). At concentrations up to 200 µg/ml, neither pro-DCD nor peg-pro-DCD-C34S reduced CFU (**Figure 5A**), suggesting that pro-DCD or peg-pro-DCD-C34S could not directly kill bacteria in septic animals. To explore alternative mechanisms by which pro-DCD might enhance bacterial clearance, we examined its effect on the activation of microtubule-associated protein 1A/1B-light chain 3 (MAP1LC3, LC3) in macrophage cultures. Pro-DCD stimulation led to the formation of LC3-positive cytoplasmic puncta (**Figure 5B**), and the conversion of LC3-I to the lipidated LC3-II form (**Figure 5C**). It raised an interesting possibility that pro-DCD may promote bacterial elimination by promoting LC3-associated phagocytosis and/or autophagy, two highly conserved processes that clear extracellular and intracellular bacteria by many phagocytes.

## Discussion

Currently, there are still no effective therapies for clinical sepsis beyond adjunctive care, such as antibiotic administration and fluid resuscitation (27–29). Therefore, it is crucial to use animal models of sepsis (30) to explore novel protective proteins that can eliminate invading pathogens and mitigate sepsis-induced dysregulated inflammatory injury. In this study, we demonstrated a beneficial role pro-DCD in an experimental model of lethal bacterial infections. On one hand, pro-DCD antigen-affinity purified pAbs exacerbated sepsis-induced systemic inflammation and tissue injury. On the other hand, recombinant pro-DCD conferred a significant protection against lethal sepsis when administered repeatedly at 2 h or 24 h post-onset. Because pro-DCD contains a Cys (C) residue at position 34 that promotes the formation of pro-DCD dimers through disulfide cross-linking, we generated a pro-DCD mutant with a Cys (C)→Ser (S) substitution at this position. The resultant pro-DCD-C34S mutant remained monomeric in solution even in the absence of any reducing agents. Given the relatively short half-life (< 24 h) of a 105-amino acid pro-DCD isomer 2 with 75% sequence homology to pro-DCD (31), we chemically conjugated water-soluble PEG side-chains to the Lys (K) residue of pro-DCD-C34S. The pegylated pro-DCD-C34S effectively rescued both male and female mice from microbial infections, even when given 24 hours post-CLP at a molar concentration five-fold lower than pro-DCD or pro-DCD-C34S. These findings mirrored previous observations that systemic administration of pro-DCD-C34S attenuated hepatic ischemia-reperfusion injury (12). Therefore, it will be important to determine whether systemic administration of pro-DCD-C34S derivatives can confer protection against lethal infections or ischemia-reperfusion injuries in clinical settings.

Currently, the exact mechanism for pro-DCD-C34S-mediated protection remains unclear but may attribute to the attenuation of sepsis-induced dysregulated inflammation and tissue injury. Indeed, pegylated pro-DCD-C34S significantly attenuated sepsis-induced accumulation of G-CSF, IL-6, KC/GRO-α, MCP-1, MIP-2/GRO-β, and sTNFRI, six surrogate markers of experimental sepsis (20, 26, 32). These findings aligned with our previous reports that pro-DCD or pro-DCD-C34S attenuated LPS- or ischemia-reperfusion-induced production of chemokines such as KC/GRO-α (11) and MIP-2/GRO-β (12). Additionally, pegylated pro-DCD-C34S significantly attenuated sepsis-induced liver injury, and markedly facilitated bacterial elimination in septic animals.

The mechanisms underlying the facilitation of bacterial elimination by pro-DCD-C34S remains an intriguing subject for future investigations. It has been proposed that the C-terminal anti-microbial domains (residue 63-110) of pro-DCD can form hexameric structures that may interact with bacterial phospholipids, leading to membrane depolarization and bacterial cell death (33–35). However, in contrast to the reported antibacterial activities of C-terminal anti-microbial domains of pro-DCD (1, 2, 23), our findings showed that pro-DCD and its PEGlation derivatives failed to directly kill *E. coli* even at concentrations up to 200 µg/ml. It remains elusive whether the presence of the pro-domain may physically hinder the formation of hexameric structures in the antimicrobial domains of pro-DCD, which might be necessary for disrupting bacterial membranes and inducing bacterial cell death (33–35).

Alternatively, pro-DCD may enhance bacterial elimination by promoting LC3-associated phagocytosis and autophagic degradation of engulfed pathogens. Indeed, macrophages and monocytes utilize two highly conserved processes, phagocytosis and a special form of autophagy termed as “xenophagy”, to eliminate extracellular pathogens and destroy intracellular organisms (36–38). Specifically, the engulfment of extracellular bacteria leads to the recruitment of lipidated LC3-II to the single-membrane phagosome, which can fuse with lysosomes, resulting in rapid acidification and destruction of the ingested organism (38). Xenophagy, on the other hand, involves the formation of a double-membrane structure called the autophagosome, which also facilitates the degradation of invasive bacteria following fusion with lysosomes (39–41), a process that share similarities with the maturation of phagosome during LC3-associated phagocytosis to destroy engulfed pathogens. Therefore, LC3 activation facilitates its recruitment to both single-membrane phagosomes and double-membranes autophagosomes during LC3-associated phagocytosis and autophagy. Consistent with a previous report that DCD-containing extracellular vesicles promoted LC3-associated phagocytosis of *E. coli* by macrophages (4), we observed that pro-DCD significantly induced LC3-II production and the formation of LC3-containing puncta in macrophage cultures. It suggests that pro-DCD may enhance bacterial elimination by promoting LC3-associated phagocytosis and autophagic degradation of engulfed pathogens. This possibility is further exemplified by previous findings that various agents capable of inducing autophagy (e.g., antibiotics, endotoxins, bedaquiline, seriniquinone, or anti-bacterial peptides) can enhance antimicrobial responses (42–47). However, future independent studies are needed to investigate whether pro-DCD loses its protective effects against lethal sepsis when LC3 is pharmacologically inhibited or genetically disrupted in experimental animals.

There are a few limitations in the current study: i) It remains elusive whether pro-DCD facilitates bacterial elimination by promoting LC3-associated phagocytosis and/or autophagic degradation of engulfed pathogens. ii) We did not assess systemic inflammatory cytokine profiles at later stages of sepsis, because many septic animals in the control group likely succumbed to the disease between 24 and 48 h post-CLP (as shown in **Figure 3D**). This made blood sampling at later time points, especially after the death of some animals in the control group, practically infeasible (22). Despite these limitations, our findings of pro-DCD as an inducible and beneficial protein have presented a promising opportunity to develop pro-DCD-C34S-based therapeutics for treating lethal microbial infections. Therefore, it is crucial to advance the development of pro-DCD-C34S derivatives and further translate our pre-clinical research into clinical treatments for bacterial infections.

## Data Availability

The original contributions presented in the study are included in the article/**Supplementary Material**. Further inquiries can be directed to the corresponding author.

## References

[1] Schittek B Hipfel R Sauer B Bauer J Kalbacher H Stevanovic S. Dermcidin: A novel human antibiotic peptide secreted by sweat glands. Nat Immunol. (2001) 2:1133–7. doi: 10.1038/ni732 11694882

[2] Schittek B. The multiple facets of dermcidin in cell survival and host defense. J Innate Immun. (2012) 4:349–60. doi: 10.1159/000336844 PMC6741627 22455996

[3] Pathak S De Souza GA Salte T Wiker HG Asjo B. Hiv induces both a down-regulation of irak-4 that impairs tlr signalling and an up-regulation of the antibiotic peptide dermcidin in monocytic cells. Scand J Immunol. (2009) 70:264–76. doi: 10.1111/j.1365-3083.2009.02299.x 19703016

[4] Li T Su X Lu P Kang X Hu M Li C. Bone marrow mesenchymal stem cell-derived dermcidin-containing migrasomes enhance lc3-associated phagocytosis of pulmonary macrophages and protect against post-stroke pneumonia. Adv Sci (Weinh). (2023) 10:e2206432. doi: 10.1002/advs.202206432 37246283 PMC10401184

[5] Corasolla Carregari V Monforte M Di Maio G Pieroni L Urbani A Ricci E. Proteomics of muscle microdialysates identifies potential circulating biomarkers in facioscapulohumeral muscular dystrophy. Int J Mol Sci. (2020) 22. doi: 10.3390/ijms22010290 PMC7795508 33396627

[6] Mancuso F Lage S Rasero J Díaz-Ramón JL Apraiz A Pérez-Yarza G. Serum markers improve current prediction of metastasis development in early-stage melanoma patients: A machine learning-based study. Mol Oncol. (2020) 14:1705–18. doi: 10.1002/1878-0261.12732 PMC7400797 32485045

[7] Kohli M Sharma SK Upadhyay V Varshney S Sengupta S Basak T. Urinary epcr and dermcidin as potential novel biomarkers for severe adult osa patients. Sleep Med. (2019) 64:92–100. doi: 10.1016/j.sleep.2019.07.002 31677485

[8] Landgraf P Sieg F Wahle P Meyer G Kreutz MR Pape HC. A maternal blood-borne factor promotes survival of the developing thalamus. FASEB J. (2005) 19:225–7. doi: 10.1096/fj.04-1789fje 15583035

[9] Esposito G Schiattarella GG Perrino C Cattaneo F Pironti G Franzone A. Dermcidin: A skeletal muscle myokine modulating cardiomyocyte survival and infarct size after coronary artery ligation. Cardiovasc Res. (2015) 107:431–41. doi: 10.1093/cvr/cvv173 26101262

[10] Li W Zhu S Li J Assa A Jundoria A Xu J. Egcg stimulates autophagy and reduces cytoplasmic hmgb1 levels in endotoxin-stimulated macrophages. Biochem Pharmacol. (2011) 81:1152–63. doi: 10.1016/j.bcp.2011.02.015 PMC3072446 21371444

[11] Wang E Qiang X Li J Zhu S Wang P. The *in vitro* immune-modulating properties of a sweat gland-derived antimicrobial peptide dermcidin. Shock. (2016) 45:28–32. doi: 10.1097/SHK.0000000000000488 26529659 PMC4684748

[12] Qiang X Li J Zhu S He M Chen W Al-Abed Y. Human dermcidin protects mice against hepatic ischemia-reperfusion-induced local and remote inflammatory injury. Front Immunol. (2021) 12:821154. doi: 10.3389/fimmu.2021.821154 35095926 PMC8795592

[13] Chen W Qiang X Wang Y Zhu S Li J Babaev A. Identification of tetranectin-targeting monoclonal antibodies to treat potentially lethal sepsis. Sci Transl Med. (2020) 12. doi: 10.1126/scitranslmed.aaz3833 PMC7169984 32295901

[14] Zhu CS Qiang X Chen W Li J Lan X Yang H. (Pcts-L)-neutralizing monoclonal antibodies to treat potentially lethal sepsis. Sci Adv. (2023) 9:eadf4313. doi: 10.1126/sciadv.adf4313 36735789 PMC9897667

[15] Li W Bao G Chen W Qiang X Zhu S Wang S. Connexin 43 hemichannel as a novel mediator of sterile and infectious inflammatory diseases. Sci Rep. (2018) 8:166–18452. doi: 10.1038/s41598-017-18452-1 29317708 PMC5760527

[16] Osuchowski MF Ayala A Bahrami S Bauer M Boros M Cavaillon JM. Minimum quality threshold in pre-clinical sepsis studies (Mqtipss): an international expert consensus initiative for improvement of animal modeling in sepsis. Shock. (2018) 50:377–80. doi: 10.1097/shk.0000000000001212 PMC6133201 30106875

[17] Zingarelli B Coopersmith CM Drechsler S Efron P Marshall JC Moldawer L. Part I: minimum quality threshold in preclinical sepsis studies (Mqtipss) for study design and humane modeling endpoints. Shock. (2019) 51:10–22. doi: 10.1097/shk.0000000000001243 30106874 PMC6296871

[18] Hellman J Bahrami S Boros M Chaudry IH Fritsch G Gozdzik W. Part iii: minimum quality threshold in preclinical sepsis studies (Mqtipss) for fluid resuscitation and antimicrobial therapy endpoints. Shock. (2019) 51:33–43. doi: 10.1097/shk.0000000000001209 29923896

[19] Chen W Zhu CS Qiang X Chen S Li J Wang P. Development of procathepsin L (Pcts-L)-inhibiting lanosterol-carrying liposome nanoparticles to treat lethal sepsis. Int J Mol Sci. (2023) 24. doi: 10.3390/ijms24108649 PMC10217857 37239992

[20] Chen W Brenner M Aziz M Chavan SS Deutschman CS Diamond B. Buprenorphine markedly elevates a panel of surrogate markers in a murine model of sepsis. Shock. (2019) 52:550–3. doi: 10.1097/SHK.0000000000001361 PMC6791512 31486774

[21] Cotroneo TM Hugunin KM Shuster KA Hwang HJ Kakaraparthi BN Nemzek-Hamlin JA. Effects of buprenorphine on a cecal ligation and puncture model in C57bl/6 mice. J Am Assoc Lab Anim Sci. (2012) 51:357–65. doi: 10.3389/fimmu.2024.1368448 PMC3358986 22776195

[22] Qiang X Chen W Zhu CS Li J Qi T Lou L. Therapeutic potential of procathepsin L-inhibiting and progesterone-entrapping dimethyl-β-cyclodextrin nanoparticles in treating experimental sepsis. Front Immunol. (2024) 15:1368448. doi: 10.3389/fimmu.2024.1368448 38550581 PMC10972846

[23] CipÃ¡kovÃ¡ I GasperÃ­k J HostinovÃ¡ E. Expression and purification of human antimicrobial peptide, dermcidin, in escherichia coli. Protein Expr Purif. (2006) 45:269–74. doi: 10.1016/j.pep.2005.07.002 16125410

[24] Heuer JG Sharma GR Gerlitz B Zhang T Bailey DL Ding C. Evaluation of protein C and other biomarkers as predictors of mortality in a rat cecal ligation and puncture model of sepsis. Crit Care Med. (2004) 32:1570–8. doi: 10.1097/01.CCM.0000129488.54282.1A 15241104

[25] Li W Ashok M Li J Yang H Sama AE Wang H. A major ingredient of green tea rescues mice from lethal sepsis partly by inhibiting hmgb1. PLoS One. (2007) 2:e1153. doi: 10.1371/journal.pone.0001153 17987129 PMC2048740

[26] Bozza FA Salluh JI Japiassu AM Soares M Assis EF Gomes RN. Cytokine profiles as markers of disease severity in sepsis: A multiplex analysis. Crit Care. (2007) 11:R49. doi: 10.1186/cc5783 17448250 PMC2206478

[27] Buchman TG Simpson SQ Sciarretta KL Finne KP Sowers N Collier M. Sepsis among medicare beneficiaries: 1. The Burdens of Sepsis, 2012-2018. Crit Care Med. (2020) 48:276–88. doi: 10.1097/CCM.0000000000004224 PMC7017943 32058366

[28] Tindal EW Armstead BE Monaghan SF Heffernan DS Ayala A. Emerging therapeutic targets for sepsis. Expert Opin Ther Targets. (2021) 25:175–89. doi: 10.1080/14728222.2021.1897107 PMC8122062 33641552

[29] Reinhart K Daniels R Kissoon N MaChado FR Schachter RD Finfer S. Recognizing sepsis as a global health priority - a who resolution. N Engl J Med. (2017) 377:414–7. doi: 10.1056/NEJMp1707170 28658587

[30] Wang H Ayala A Aziz M Billiar TR Deutschman CS Jeyaseelan S. Value of animal sepsis research in navigating the translational labyrinth. Front Immunol. (2025) 16:1593342. doi: 10.3389/fimmu.2025.1593342 40303397 PMC12037402

[31] Ghosh R Karmohapatra SK Bhattacharyya M Bhattacharya R Bhattacharya G Sinha AK. The appearance of dermcidin isoform 2, a novel platelet aggregating agent in the circulation in acute myocardial infarction that inhibits insulin synthesis and the restoration by acetyl salicylic acid of its effects. J Thromb Thrombolysis. (2011) 31:13–21. doi: 10.1007/s11239-010-0515-z 20809104

[32] Osuchowski MF Welch K Siddiqui J Remick DG. Circulating cytokine/inhibitor profiles reshape the understanding of the sirs/cars continuum in sepsis and predict mortality. J Immunol. (2006) 177:1967–74. doi: 10.4049/jimmunol.177.3.1967 16849510

[33] Song C Weichbrodt C Salnikov ES Dynowski M Forsberg BO Bechinger B. Crystal structure and functional mechanism of a human antimicrobial membrane channel. Proc Natl Acad Sci U S A. (2013) 110:4586–91. doi: 10.1073/pnas.1214739110 PMC3607029 23426625

[34] Burian M Schittek B. The secrets of dermcidin action. Int J Med Microbiol. (2015) 305:283–6. doi: 10.1016/j.ijmm.2014.12.012 25596890

[35] Zeth K Sancho-Vaello E. The human antimicrobial peptides dermcidin and ll-37 show novel distinct pathways in membrane interactions. Front Chem. (2017) 5:86. doi: 10.3389/fchem.2017.00086 29164103 PMC5681987

[36] Nakagawa I Amano A Mizushima N Yamamoto A Yamaguchi H Kamimoto T. Autophagy defends cells against invading group a streptococcus. Science. (2004) 306:1037–40. doi: 10.1126/science.1103966 15528445

[37] Gutierrez MG Master SS Singh SB Taylor GA Colombo MI Deretic V. Autophagy is a defense mechanism inhibiting bcg and mycobacterium tuberculosis survival in infected macrophages. Cell. (2004) 119:753–66. doi: 10.1016/j.cell.2004.11.038 15607973

[38] Sanjuan MA Dillon CP Tait SW Moshiach S Dorsey F Connell S. Toll-like receptor signalling in macrophages links the autophagy pathway to phagocytosis. Nature. (2007) 450:1253–7. doi: 10.1038/nature06421 18097414

[39] Alonso S Pethe K Russell DG Purdy GE. Lysosomal killing of mycobacterium mediated by ubiquitin-derived peptides is enhanced by autophagy. Proc Natl Acad Sci U S A. (2007) 104:6031–6. doi: 10.1073/pnas.0700036104 PMC1851611 17389386

[40] Mizushima N. The pleiotropic role of autophagy: from protein metabolism to bactericide. Cell Death Differ. (2005) 12:1535–41. doi: 10.1038/sj.cdd.4401728 16247501

[41] Checroun C Wehrly TD Fischer ER Hayes SF Celli J. Autophagy-Mediated Reentry of Francisella Tularensis into the Endocytic Compartment after Cytoplasmic Replication. Proc Natl Acad Sci U S A. (2006) 103:14578–83. doi: 10.1073/pnas.0601838103 PMC1600002 16983090

[42] Huang J Ita M Zhou H Zhao H Hassan F Bai Z. Autophagy induced by taurolidine protects against polymicrobial sepsis by promoting both host resistance and disease tolerance. Proc Natl Acad Sci U S A. (2022) 119:e2121244119. doi: 10.1073/pnas.2121244119 35512102 PMC9171638

[43] Kim JJ Lee HM Shin DM Kim W Yuk JM Jin HS. Host cell autophagy activated by antibiotics is required for their effective antimycobacterial drug action. Cell Host Microbe. (2012) 11:457–68. doi: 10.1016/j.chom.2012.03.008 22607799

[44] Wang J Feng X Zeng Y Fan J Wu J Li Z. Lipopolysaccharide (Lps)-induced autophagy is involved in the restriction of escherichia coli in peritoneal mesothelial cells. BMC Microbiol. (2013) 13:255. doi: 10.1186/1471-2180-13-255 24219662 PMC3833177

[45] Giraud-Gatineau A Coya JM Maure A Biton A Thomson M Bernard EM. The antibiotic bedaquiline activates host macrophage innate immune resistance to bacterial infection. Elife. (2020) 9. doi: 10.7554/eLife.55692 PMC7200153 32369020

[46] Trzoss L Fukuda T Costa-Lotufo LV Jimenez P La Clair JJ Fenical W. Seriniquinone, a selective anticancer agent, induces cell death by autophagocytosis, targeting the cancer-protective protein dermcidin. Proc Natl Acad Sci U S A. (2014) 111:14687–92. doi: 10.1073/pnas.1410932111 PMC4205641 25271322

[47] Peláez Coyotl EA Barrios Palacios J Muciño G Moreno-Blas D Costas M Montiel Montes T. Antimicrobial peptide against mycobacterium tuberculosis that activates autophagy is an effective treatment for tuberculosis. Pharmaceutics. (2020) 12. doi: 10.3390/pharmaceutics12111071 PMC7697726 33182483

