# Supplementary material for: Pro-dermcidin and derivatives as potential therapeutics for lethal experimental sepsis
Source: Front Immunol. 2025 Jun 4;16:1621633. doi: 10.3389/fimmu.2025.1621633 (PMC12174047; doi:10.3389/fimmu.2025.1621633)
Supplement: Supplementary file 1 [file DataSheet1.docx]

**Supplementary Materials for**

**Identification of pro-dermcidin and derivatives as potential therapeutics for lethal experimental sepsis.**

Weiqiang Chen^1, 2#^, Xiaoling Qiang^1, 2#^, Cassie Shu Zhu^1, 2#^, Jianhua Li^1^, Li Lou^1^, Ping Wang^1, 2^, Kevin J. Tracey^1, 2^, and Haichao Wang^1, 2*^

**^#^** equally contributed

**^*^** Corresponding author: E-mail: [hwang@northwell.edu](mailto:hwang@northwell.edu)

**Materials and Methods**

**Western blotting**

The concentrations of pro-DCD in human PBMC-conditioned culture medium were determined by Western blotting analysis using either commercial polyclonal antibodies (Cat.# SC27467, Santa Cruz) or home-made rabbit anti-pro-DCD polyclonal antibodies. Briefly, equal volume of cell-conditioned culture medium or murine/human serum were resolved on sodium dodecyl sulfate (SDS)-polyacrylamide gels and transferred to polyvinylidene difluoride (PVDF) membranes. After blocking with 5% nonfat milk, the membranes were incubated with the appropriate antibodies (anti-pro-DCD, 1:1000) overnight. Subsequently, the membranes were incubated with secondary antibodies (donkey-anti-rabbit IgG-HRP, Cat. # NA934, GH Healthcare), and the immune-reactive bands were visualized by chemiluminescence.

**Figure S1**

**Figure 1S. Western blotting analysis of our home-made pro-DCD-C34S using commercial DCD-reactive polyclonal antibodies.** Home-made recombinant pro-DCD-C34S and bacterial endotoxins were separated on SDS-PAGE gels and immunoblotted with commercial polyclonal antibodies (Cat. # ab175519, Abcam) targeting a unique peptide corresponding to residue 96-110 of human pro-DCD.

**Table S1. Reagent sources**

|  | SOURCE | IDENTIFIER |
| --- | --- | --- |
| **Antibodies** | | |
| Mouse anti-β-actin antibody | Sigma-Aldrich | Cat. # A1978 |
| Rabbit anti-human DCD polyclonal antibodies (residue 96-110) | Abcam | Cat. # ab175519 |
| Rabbit anti-human DCD polyclonal antibodies (C-terminus) | Santa Cruz | Cat. # sc27467 |
| Rabbit anti-mouse LC3A/B monoclonal antibodies | Cell Signaling | Cat. # 12741 |
| HRP-conjugated mouse anti-rabbit IgG | Santa Cruz | Cat, # sc2357 |
| HRP conjugated donkey anti-rabbit IgG | GE Healthcare | Cat. # NA934 |
| **Chemicals, Peptides, and Recombinant Proteins** |  |  |
| Crude bacterial endotoxin (lipopolysaccharide, LPS) | Sigma-Aldrich | *E. coli 0111:B4* |
| Human serum | Sigma-Aldrich | Cat. # H3667 |
| Methyl-PEG_24_-NHS ester | Thermo Scientific | Cat. # 22687 |
| Cyanogen bromide (CNBr)-activated Sepharose4 agarose beads | GE Healthcare | Cat. # 17098101 |
| Protein A/G Sepharose® | Abcam | Cat. # ab193262 |
| Dulbecco’s modified Eagle medium (DMEM) | Invitrogen/Life Technologies | Cat. # 11995-065 |
| OPTI-MEM I Reduced-Serum Medium | ThermoFisher Scientific | Cat. # 31985062 |
| Penicillin/streptomycin | Invitrogen/Life Technologies | Cat. # 15140-122 |
| **Enzyme Assays, Cytokine Antibody Arrays** |  |  |
| Aspartate Aminotransferase (AST) assay | Pointe Scientific | Cat. # 7561 |
| Alanine Aminotransferase (ALT) assay | Pointe Scientific | Cat. #7526 |
|  |  |  |
| Murine Cytokine Antibody Arrays | RayBiotech Inc | Cat. #. M0308003 |
| Human Cytokine Antibody C3 Arrays | RayBiotech Inc | Cat. # AAH-CYT-3-4 |
| **Mice** |  |  |
| Balb/C mice | Jackson Laboratory | Stock # 000651 |
| **Software and Algorithms** |  |  |
| UN-SCAN-IT Gel Analysis Software Version 7.1 | Silk Scientific Inc. |  |
